# Complete Recovery After a Witnessed Ventricular Fibrillation Arrest: A Case Report

**DOI:** 10.7759/cureus.105334

**Published:** 2026-03-16

**Authors:** Hüseyin Aldemir

**Affiliations:** 1 Emergency Department, Afyonkarahisar State Hospital, Afyonkarahisar, TUR

**Keywords:** acute myocardial infarction-cardiac arrest, idiopathic ventricular fibrillation, in-hospital cardiac arrest, post-arrest care, st-elevation myocardial infarction (stemi), sudden cardiac arrest, ventricular fibrillation arrest

## Abstract

Sudden cardiac arrest remains one of the leading causes of mortality worldwide and is frequently the first manifestation of previously undiagnosed coronary artery disease. Early defibrillation and rapid implementation of advanced cardiac life support are the most critical determinants of survival and neurological recovery. We present the case of a 52-year-old male with no known comorbidities, but a significant smoking history (30 pack-years), who presented with typical chest pain and developed witnessed ventricular fibrillation arrest in the emergency department. Immediate defibrillation according to current resuscitation guidelines resulted in return of spontaneous circulation within five minutes, with full neurological recovery (Glasgow Coma Scale score of 15). Coronary angiography revealed a 100% occlusion of the circumflex artery, successfully treated with primary percutaneous coronary intervention. This case illustrates the critical importance of early defibrillation, adherence to resuscitation algorithms, trauma evaluation following collapse, and rapid access to definitive coronary reperfusion. This case demonstrates the synergy between emergency trauma protocols and rapid cardiac intervention. This case further emphasizes that while rapid clinical success was achieved in a controlled setting, the outcome would likely be less favorable in out-of-hospital environments, highlighting the urgent need for widespread public access to automated external defibrillators and the strengthening of community-based survival chains as key preventive measures.

## Introduction

Sudden cardiac arrest (SCA) remains one of the leading causes of death globally [1-3]. Acute coronary syndrome (ACS) is the most common precipitating factor, frequently triggering malignant ventricular arrhythmias such as ventricular fibrillation (VF) [1,4,5]. According to the guidelines of the European Society of Cardiology (ESC), American Heart Association (AHA), and European Resuscitation Council (ERC), rapid defibrillation in shockable rhythms is the most effective intervention to improve survival and neurological outcomes [1-3]. The “Chain of Survival,” first conceptualized by the AHA, emphasizes early recognition, immediate high-quality cardiopulmonary resuscitation (CPR), early defibrillation, advanced life support, and post-resuscitation care [2]. Witnessed in-hospital arrests offer a significantly better prognosis due to minimal delay in initiating resuscitative measures [2,6,7]. Epidemiological data indicate that in-hospital cardiac arrests (IHCAs) occurring with shockable rhythms, such as VF, carry a significantly higher survival-to-discharge rate, ranging from 25% to over 50%, when compared to out-of-hospital events, provided that the “Chain of Survival” is activated immediately [2,6,7]. While inferolateral ST-segment elevation is frequently associated with right coronary artery (RCA) occlusion, circumflex (Cx) artery occlusion often presents with similar patterns, potentially leading to significant diagnostic challenges and sudden electrical instability [4,5]. IHCAs with shockable rhythms carry a significantly higher discharge survival rate compared to out-of-hospital events, emphasizing the “Chain of Survival” efficiency. While Cx artery occlusions are notorious for “silent” or atypical ECG presentations, they require the same urgency as left anterior descending (LAD) artery lesions.

## Case presentation

A 52-year-old male with a 30-pack-year smoking history and no known chronic disease presented to the emergency department with typical retrosternal chest pain that began shortly after returning home from work. On admission, vital signs were within normal limits, the Glasgow Coma Scale (GCS) score was 15, and he was hemodynamically stable. While being prepared for a 12-lead ECG in the observation area, the patient suddenly lost consciousness and fell from his own standing height, striking the frontal region of his head.

Defibrillator pads were applied within the first minute, the rhythm was analyzed as VF, and a biphasic shock of 200 J was delivered. Advanced cardiac life support was initiated without delay in accordance with current resuscitation guidelines. High-quality CPR was performed, and three biphasic defibrillation shocks (200 J, 200 J, 270 J) were delivered. The patient received 300 mg intravenous amiodarone and 2 mg (two times 1 mg, every three minutes) intravenous adrenaline during resuscitation. Return of spontaneous circulation (ROSC) was achieved after approximately five minutes of high-quality cardiopulmonary resuscitation. Following ROSC, the patient regained full consciousness with a GCS score of 15, was cooperative and oriented, and remained hemodynamically stable. As the patient was adequately ventilated with a bag-valve mask throughout the entire intervention and achieved immediate, full neurological recovery with a return of protective airway reflexes upon ROSC, endotracheal intubation was not required.

**Figure 1:**
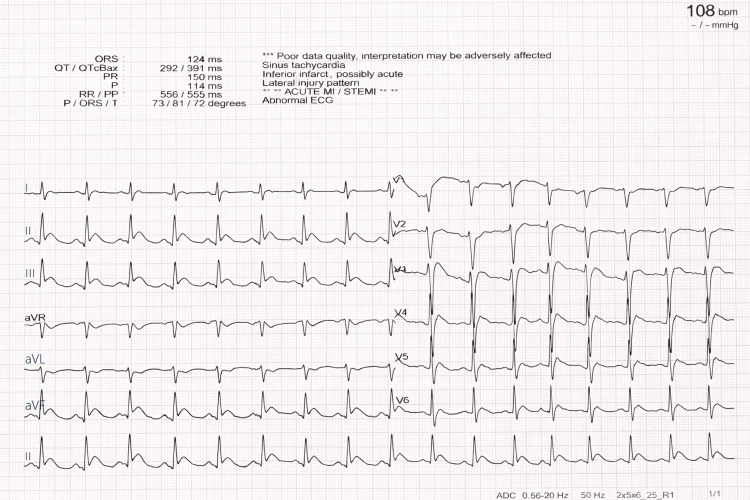
Post-return of spontaneous circulation 12-lead ECG revealing inferolateral ST-segment elevation, consistent with an acute inferolateral ST-elevation myocardial infarction.

The post-ROSC ECG demonstrated inferolateral ST-segment elevation consistent with ST-elevation myocardial infarction (STEMI) (Figure 1).

Physical examination revealed a 5 × 5 cm abrasion over the frontal midline region consistent with impact from a fall at ground level. Neurological examination showed no focal deficits. Non-contrast cranial CT was performed immediately post-ROSC to evaluate the trauma sustained during the fall. The results demonstrated no evidence of acute intracranial hemorrhage, skull fracture, or space-occupying lesions. Neurosurgical consultation did not recommend emergent intervention. Close neurological monitoring was advised, with repeat cranial imaging only if clinical deterioration occurred. No contraindication to dual antiplatelet therapy or urgent STEMI management was identified. The patient was loaded with 300 mg of aspirin and 600 mg of clopidogrel and transferred to the catheterization laboratory after informed consent was obtained directly from him. The total time from emergency department admission to the initiation of coronary angiography was 16 minutes.

**Figure 2:**
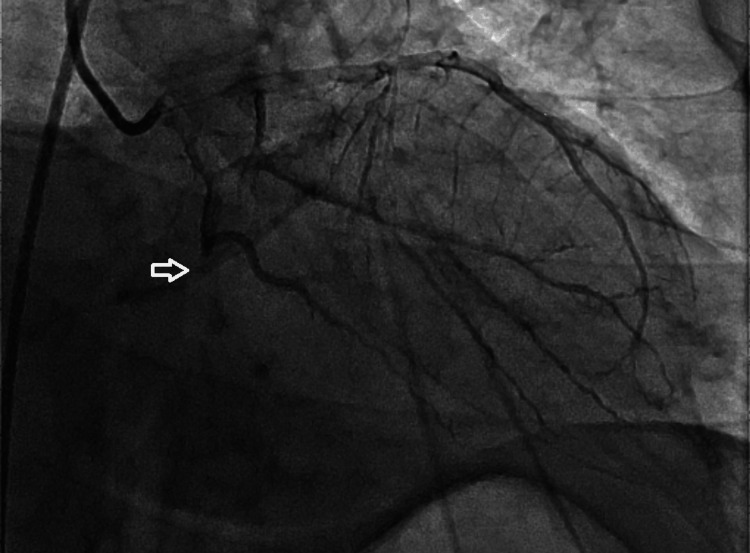
Pre-interventional coronary angiography demonstrating a 100% total occlusion of the circumflex (Cx) artery (arrow).

**Figure 3:**
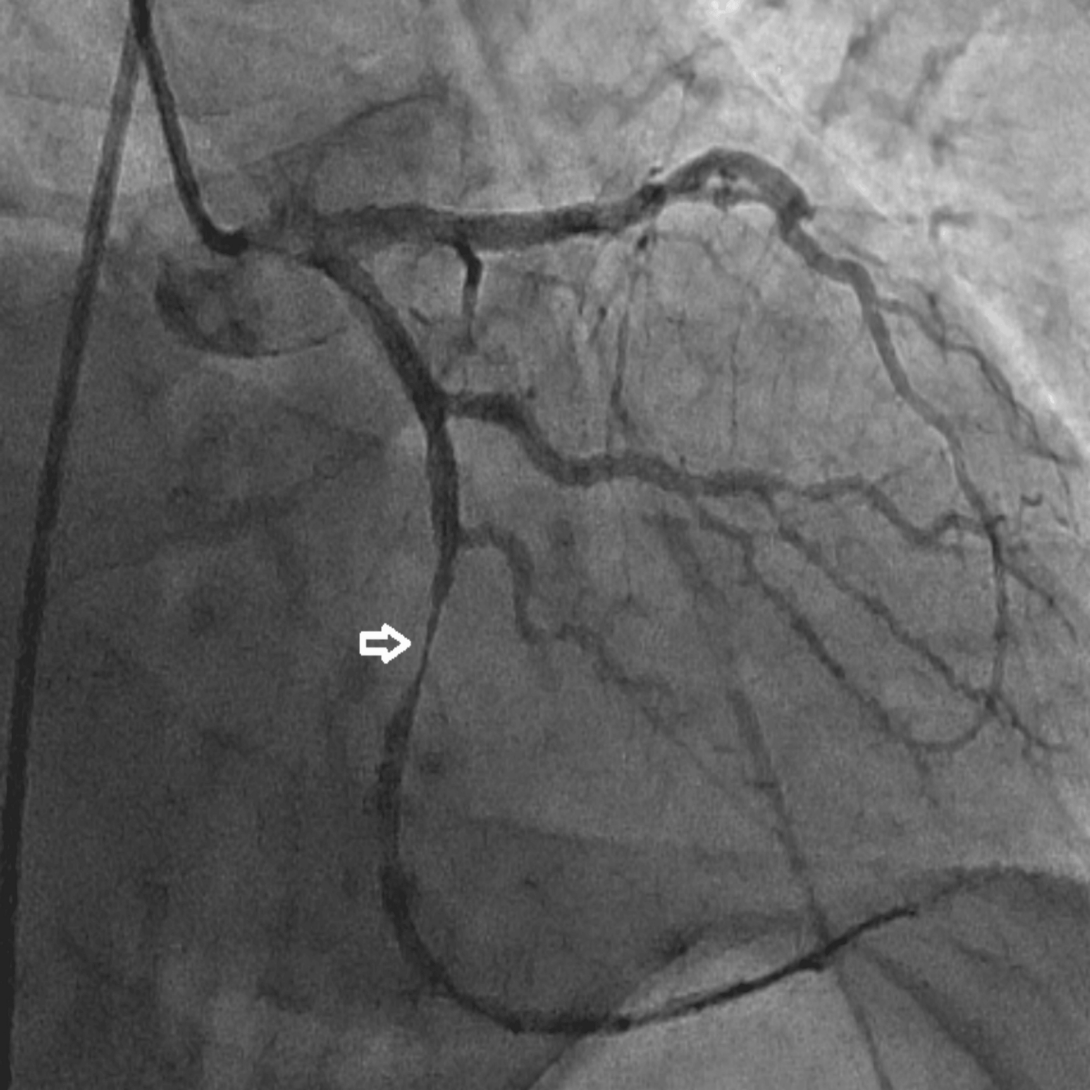
Intraprocedural coronary angiography showing successful crossing of the culprit lesion in the circumflex (Cx) artery (arrow) with a floppy guidewire followed by balloon predilatation.

**Figure 4:**
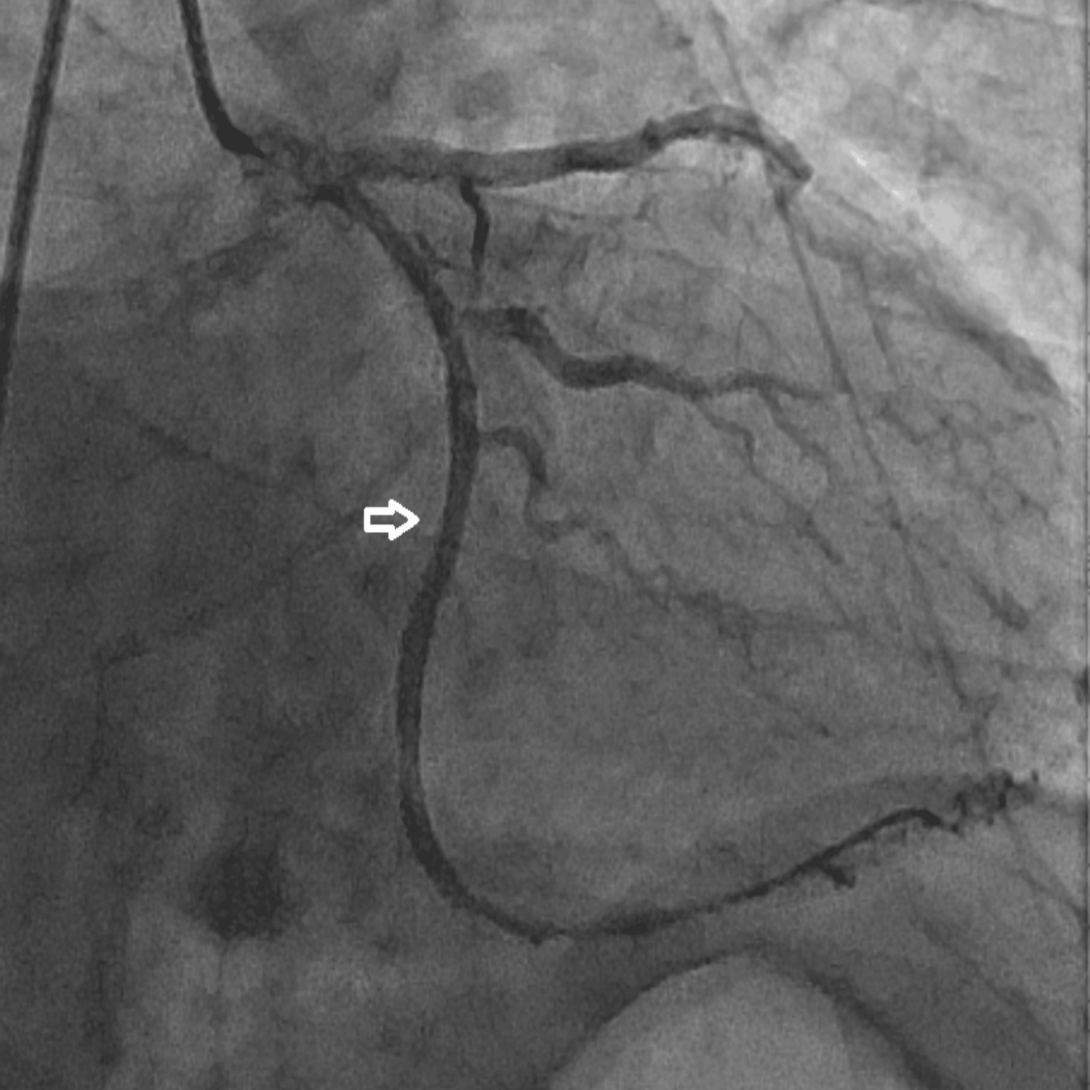
Post-interventional coronary angiography after implantation of a drug-eluting stent, demonstrating complete restoration of coronary blood flow with Thrombolysis in Myocardial Infarction 3 flow (arrow).

Coronary angiography revealed atherosclerotic plaques in the LAD and RCAs and a 100% total occlusion of the Cx artery. The lesion was successfully crossed with a floppy guidewire, predilatation was performed using a 2.0 × 12 mm balloon, and a 3.0 × 24 mm drug-eluting stent was implanted. Final angiography confirmed complete restoration of normal coronary blood flow, achieving a Thrombolysis in Myocardial Infarction (TIMI) 3 flow grade. The angiographic sequence is shown in Figures 2-4.

The procedure was completed without complication, and the patient was admitted to the coronary intensive care unit in stable condition with preserved neurological function. The patient remained in the coronary intensive care unit for 36 hours for close hemodynamic and neurological monitoring, followed by an additional 36 hours in the cardiology ward for further stabilization.

The patient was discharged uneventfully without neurological deficit or procedural complications and was considered to have achieved complete clinical recovery. At discharge, he was prescribed dual antiplatelet therapy, statins, and beta-blockers. He was also referred to a smoking cessation clinic and received counseling on lifestyle modifications to reduce future cardiovascular risk.

## Discussion

This case illustrates the decisive impact of immediate defibrillation and uninterrupted high-quality CPR in shockable cardiac arrest rhythms. As emphasized in the guidelines of the ESC, AHA, and ERC, rapid rhythm recognition and early defibrillation are the strongest predictors of survival in VF [1-3,6]. The witnessed nature of the arrest and the minimal delay before defibrillation likely limited cerebral hypoxia time, explaining the patient’s complete neurological recovery despite a sudden circulatory collapse [2,7].

Although inferolateral ST-segment elevation typically suggests a dominant RCA or Cx artery occlusion, the precise culprit lesion can be difficult to predict due to variations in coronary dominance and posterior extension [5]. This reinforces the importance of urgent coronary angiography in post-arrest STEMI patients regardless of presumed culprit vessel based solely on surface ECG findings [4-6].

Another important aspect of this case is the structured evaluation following the collapse. Even in the presence of STEMI requiring urgent reperfusion, assessment for traumatic injury remains essential [8]. The absence of acute pathology on cranial CT and neurosurgical recommendation for close observation without emergent intervention allowed safe initiation of dual antiplatelet therapy and expedited transfer for primary PCI without delay [6,8]. The absence of contraindication to antithrombotic therapy ensured adherence to guideline-directed STEMI management without compromising patient safety [2-4]. Furthermore, achieving complete restoration of coronary blood flow, as defined by TIMI 3 flow grade, is a critical procedural endpoint that correlates with improved myocardial salvage and long-term clinical outcomes in patients with acute coronary occlusion [4,9].

The coordinated response between emergency and cardiology teams, rapid implementation of resuscitation protocols, immediate neurological assessment, and prompt primary PCI represent a practical application of the modern “Chain of Survival” [1-3]. This integrated approach resulted in survival with intact neurological function in a patient who experienced sudden VF arrest as the first manifestation of acute coronary occlusion [1,4-6].

## Conclusions

Acute occlusion of the Cx artery may present with atypical clinical findings and can rapidly deteriorate into life-threatening VF. This case illustrates that witnessed VF cardiac arrest secondary to acute coronary occlusion can result in complete neurological recovery when early rhythm recognition and immediate defibrillation are promptly delivered in accordance with resuscitation guidelines. Rapid initiation of advanced cardiac life support and timely primary PCI were critical in restoring coronary perfusion and achieving a favorable outcome. In addition, the case highlights the importance of simultaneously evaluating and managing potential traumatic injuries following sudden collapse, emphasizing the need for coordinated emergency, trauma, and interventional cardiology care in patients presenting with cardiac arrest related to ACS.

## References

[1] Zeppenfeld K, Tfelt-Hansen J, de Riva M (2022). 2022 ESC Guidelines for the management of patients with ventricular arrhythmias and the prevention of sudden cardiac death. Eur Heart J.

[2] Panchal AR, Bartos JA, Wyckoff MH (2025). Part 2: evidence evaluation and guidelines development: 2025 American Heart Association Guidelines for Cardiopulmonary Resuscitation and Emergency Cardiovascular Care. Circulation.

[3] Soar J, Böttiger BW, Carli P (2021). European Resuscitation Council Guidelines 2021: adult advanced life support. Resuscitation.

[4] Byrne RA, Rossello X, Coughlan JJ (2023). 2023 ESC Guidelines for the management of acute coronary syndromes. Eur Heart J.

[5] Birnbaum Y, Wilson JM, Fiol M, de Luna AB, Eskola M, Nikus K (2014). ECG diagnosis and classification of acute coronary syndromes. Ann Noninvasive Electrocardiol.

[6] Nolan JP, Sandroni C, Böttiger BW (2021). European Resuscitation Council and European Society of Intensive Care Medicine Guidelines 2021: post-resuscitation care. Resuscitation.

[7] Andersen LW, Holmberg MJ, Berg KM, Donnino MW, Granfeldt A (2019). In-hospital cardiac arrest: a review. JAMA.

[8] Rapp A, Kobeissi H, Fahim DK (2025). Updated review of the management of and guidelines for traumatic brain injury. J Clin Med.

[9] Gibson CM, Cannon CP, Murphy SA, Marble SJ, Barron HV, Braunwald E (2002). Relationship of the TIMI myocardial perfusion grades, flow grades, frame count, and percutaneous coronary intervention to long-term outcomes after thrombolytic administration in acute myocardial infarction. Circulation.

